# Advanced Fault-Tolerant Anti-Surge Control System of Centrifugal Compressors for Sensor and Actuator Faults

**DOI:** 10.3390/s22103864

**Published:** 2022-05-19

**Authors:** Turki Alsuwian, Arslan Ahmed Amin, Muhammad Taimoor Maqsood, Muhammad Bilal Qadir, Saleh Almasabi, Mohammed Jalalah

**Affiliations:** 1 Department of Electrical Engineering, College of Engineering, Najran University, Najran 11001, Saudi Arabia; tmalsuwian@nu.edu.sa (T.A.); ssalmasabi@nu.edu.sa (S.A.); msjalalah@nu.edu.sa (M.J.); 2 Department of Electrical Engineering, FAST National University of Computer and Emerging Sciences, Chiniot 35400, Pakistan; f180858@nu.edu.pk; 3 School of Engineering & Technology, National Textile University, Faisalabad 37610, Pakistan; bilal.qadir@ntu.edu.pk; 4 Promising Centre for Sensors and Electronic Devices (PCSED), Advanced Materials and Nano-Research Centre, Najran University, P.O. Box 1988, Najran 11001, Saudi Arabia

**Keywords:** fault-tolerant control, anti-surge control, compressor control, redundancy, reliability, centrifugal compressors

## Abstract

Faults frequently occur in the sensors and actuators of process machines to cause shutdown and process interruption, thereby creating costly production loss. centrifugal compressors (CCs) are the most used equipment in process industries such as oil and gas, petrochemicals, and fertilizers. A compressor control system called an anti-surge control (ASC) system based on many critical sensors and actuators is used for the safe operation of CCs. In this paper, an advanced active fault-tolerant control system (AFTCS) has been proposed for sensor and actuator faults of the anti-surge control system of a centrifugal compressor. The AFTCS has been built with a dedicated fault detection and isolation (FDI) unit to detect and isolate the faulty part as well as replace the faulty value with the virtual redundant value from the observer model running in parallel with the other healthy sensors. The analytical redundancy is developed from the mathematical modeling of the sensors to provide estimated values to the controller in case the actual sensor fails. Dual hardware redundancy has been proposed for the anti-surge valve (ASV). The simulation results of the proposed Fault-tolerant control (FTC) for the ASC system in the experimentally validated CC HYSYS model reveal that the system continued to operate in the event of faults in the sensors and actuators maintaining system stability. The proposed FTC for the ASC system is novel in the literature and significant for the process industries to design a highly reliable compressor control system that would continue operation despite faults in the sensors and actuators, hence preventing costly production loss.

## 1. Introduction

### 1.1. Fault-Tolerant Control Systems

Faults can occur in the field, often due to machinery errors, human errors, emergency problems, worn-out material, and other reasons. A fault is described as an abnormal deviation of a parameter from the acceptable value. Faults can cause the shutdown of the process machines, creating costly production loss, and must be prevented such that the machines should continue to operate reliably even in faulty conditions within safety limits [1]. Fault-tolerant control (FTC) is an advanced control system technique in which the system will continue operation without failure despite faults in its components such as sensors or actuators. These controls are usually implemented in automated vehicles [2] and aircraft flight safety systems [3].

### 1.2. Types of Fault-Tolerant Control Systems

**Figure 1:**
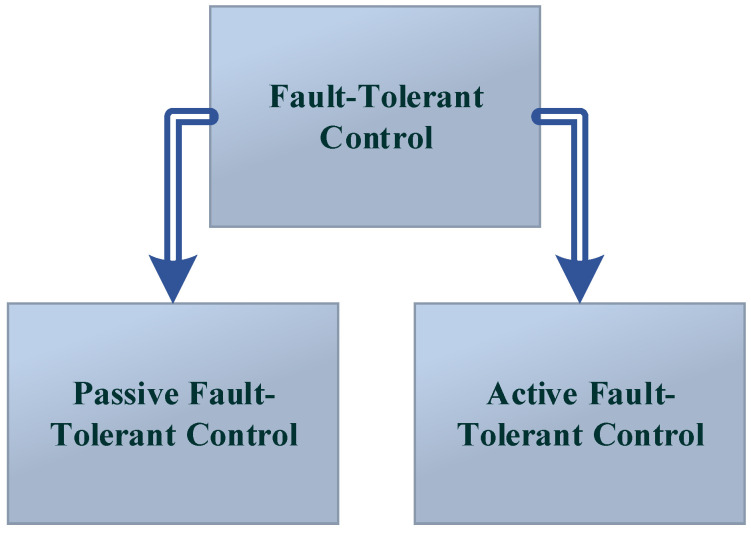
The fault-tolerant control types.

The passive fault-tolerant control system (PFTCS) and the active fault-tolerant control system (AFTCS) are the two main types of FTC [1]. These are implemented and described by many authors in [1,4,5,6,7] as mentioned in Figure 1.

The list of abbreviations and a list of symbols are mentioned in Abbreviation.

**Figure 2:**
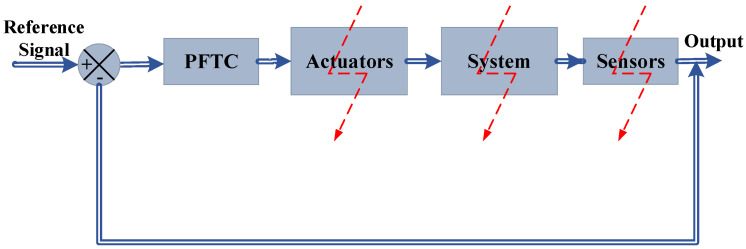
The passive fault-tolerant control [1].

In PFTCS, the fault is tolerated in such a way that the controller is robust to certain parameters and provides robustness despite the faults being occurred [8]. The implementation of such a control is executed in [9,10,11]. The PFTCS block diagram is shown in Figure 2.

**Figure 3:**
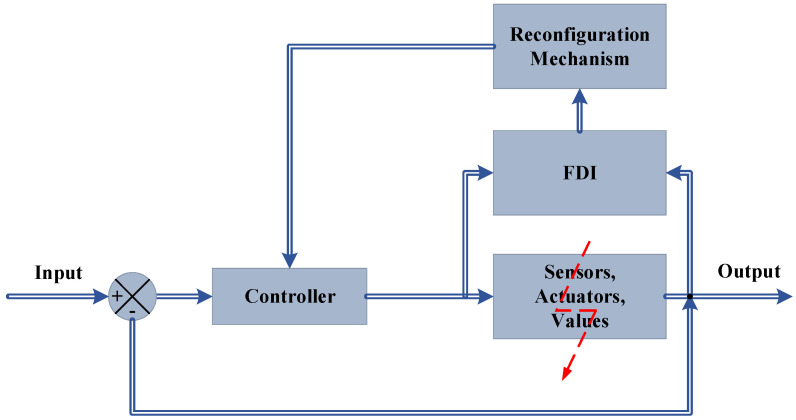
The active fault-tolerant control [1].

In the AFTCS, the fault is detected and isolated by the fault detection and isolation (FDI) Unit, after which the reconfiguration of the controller is carried out [12,13]. The block diagram for the AFTCS is depicted in Figure 3.

The PFTCS tends to be faster than the AFTCS as it contains fewer steps than the AFTCS. The delay is mainly caused by the calculations that are made in the AFTCS, which are more complex than in the PFTCS. Therefore, the PFTCS has an advantage over the AFTCS in terms of being a quicker controller than the AFTCS but is only limited to the tolerance of faults considered at the design stage This issue can be resolved if both controllers operate in parallel, forming a hybrid controller. The hybrid FTC is reviewed in [14,15,16].

### 1.3. Analytical and Hardware Redundancy

The working of more than one component in parallel to the execution of a similar function is known as redundancy, which is the key part of the design of an FTC system. During the fault occurrence in any of the two parts, the backup equipment comes into action, which helps the system to continue its operation without any disruption. It can be further divided into two categories, namely, analytical and hardware.

**Figure 4:**
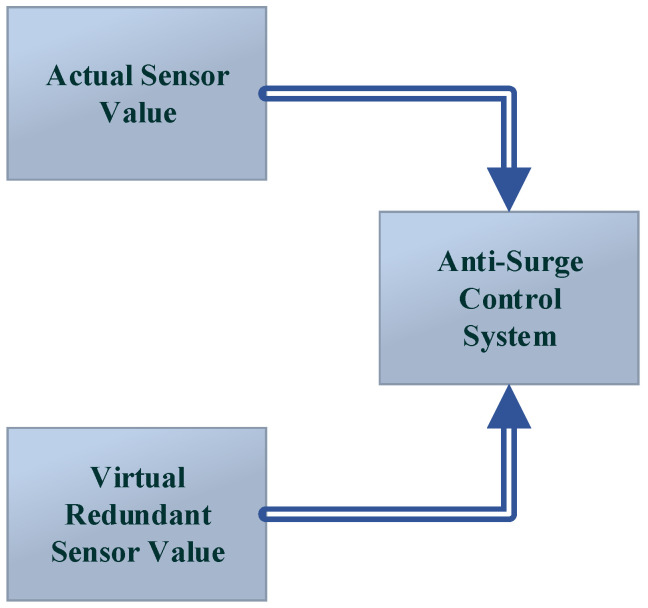
The analytical redundancy.

In analytical redundancy, the fault is accommodated by an estimated value taken from the software, which is used by the controller, while in hardware redundancy, duplicate hardware is installed in parallel to the primary hardware. Hardware redundancy is not as economical as analytical redundancy because additional hardware is attached to it, which increases the cost. Analytical redundancy is economical and is highly dependent on the accuracy of the system model. A general sketch for analytical redundancy is shown in Figure 4.

**Figure 5:**
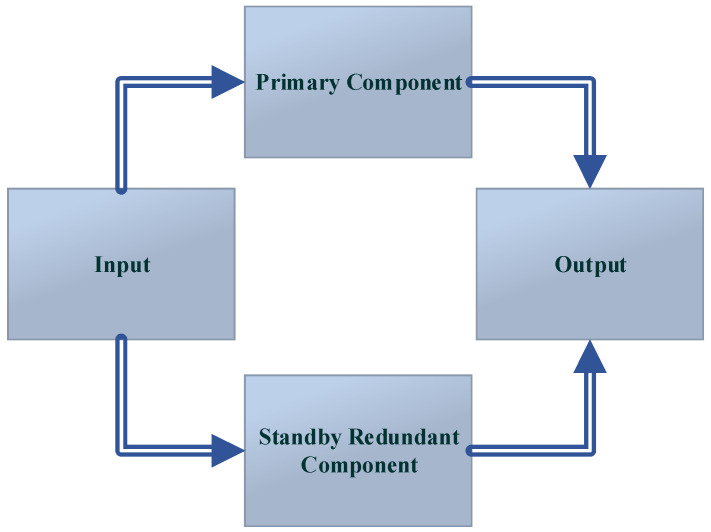
The dual redundancy [1].

The system’s reliability is increased by the hardware redundancy as it removes the fault in the primary equipment by replacing it with secondary equipment. The number of components installed in parallel, for the hardware redundancy, determines its type. If two components are installed, then it is called dual redundancy, as shown in Figure 5. Adding more components in parallel is also possible, in which triple modular redundancy (TMR) is a popular technique, but it increases the overall cost, space, and weight of the system and is recommended only after a proper cost/benefit analysis [7].

Fault-diagnostic and fault-tolerant control approaches, as well as their applications in a range of engineering systems, have made promising advancements throughout the previous four decades. The survey papers [17,18,19] provided a complete overview of real-time fault diagnosis and fault-tolerant control, with a focus on recent findings. In these works, various fault-detection methodologies and applications are thoroughly examined from both a model-based and a signal-based viewpoint. These are further examined from the knowledge-based and hybrid/active perspectives, respectively. These papers also provide comments on the benefits and drawbacks of different diagnostic strategies. The major goals of the article [20] are to use analytical redundancy relations to generate residual generation for sensor failure detection and steam generator isolation. These relationships are utilized to discover and isolate sensor faults by eliminating the system’s unknown factors. Through the individual observability of each sensor, the nonlinear system of a steam generator is linearized around the operational point and linear analytical redundancy relations are produced using the parity space approach.

### 1.4. Compressor Control System

**Figure 6:**
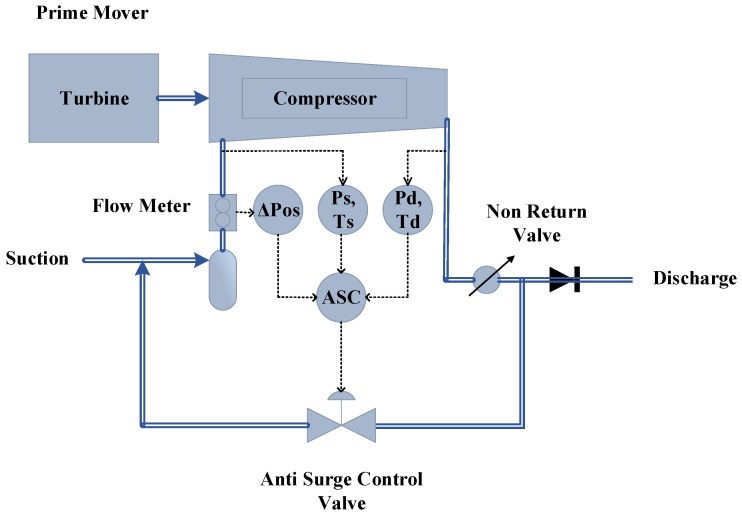
The anti-surge control [21].

The application of FTC in the process machines is a remarkable strategy to prevent their shutdown in the event of faults in the system’s components. This will result in increased reliability, safety, and reduced downtime, eventually increasing the production profits. Compressors are one of the important production machines widely used for various applications. To transport gas from remote wellhead places through pipelines to the industrial and commercial regions, the pressure needs to be increased. Hence, centrifugal compressors (CCs) are used as application output for high-pressure gas. The gas compression assembly contains coolers, pipes, scrubbers, a turbo-compressor unit, and control valves. [21] The particles of dust are initially filtered out and separated from the gas. After the filtration of the gas, the moisture and water droplets are removed, considering the compressor’s protection against liquids. The compressed gas at high temperatures is then cooled to a lower temperature with the aid of coolers. The general assembly of the compressor can be seen in Figure 6.

The anti-surge control of the compressor is the most critical controller of the compressor as the surge in the system can cause a lot of damage to the machinery and different scenarios are responsible for the occurrence of the surge. Low suction pressure, high discharge pressure, emergency shutdown, and initial start-up of the compressor are the potentially responsible conditions for the surge to occur [21].

The complete compressor model can be elaborated by (1).

$$\dot{\psi }=\frac{1}{4B^{2}l_{c}}\left(\phi -\phi_{T}\left(\psi \right)+d_{\phi }\left(\xi \right)\right)$$

(1) $$\dot{\phi }=\frac{1}{l_{c}}\left(\psi_{c}\left(\phi \right)-\psi -\psi_{V}\left(\phi \right)+d_{\psi }\left(\xi \right)\right)$$

where ψ denotes the pressure rise of the compressor and ϕ represents mass flow. ξ is dimensionless time, while $\phi_{T}\left(\psi \right)$ represents throttle valve characteristics and $\psi_{c}\left(\phi \right)$ denotes the compressor’s characteristics. $l_{c}$ denotes ducts’ and compressors’ lengths. $d_{\phi }\left(\xi \right)$ is the disturbance in the compressor flow while, $d_{\psi }\left(\xi \right)$ is the disturbance in the pressure of the compressor [22,23].

$\psi_{c}\left(\phi \right)$ is described in (2).

(2) $$\psi_{c}\left(\phi \right)=\psi_{c0}+H\left(1+\frac{3}{2}\left(\frac{\phi }{W}-1\right)-\frac{1}{2}{\left(\frac{\phi }{W}-1\right)}^{3}\right)$$

Here, $\psi_{c0}$ represents the characteristic curve value in 0 dB. The half-width and half-height are abbreviated as *W* and *H*, respectively.

Similarly, $\phi_{T}\left(\psi \right)$ is described in (3).

(3) $$\phi_{T}\left(\psi \right)=\gamma_{T}\sqrt{\psi }$$

$\gamma_{T}\sqrt{\psi }$ represents the yield of the valve.

The Greitzer parameter *B* is obtained using (4) [24,25].

(4) $$B=\frac{U}{2a_{s}}\sqrt{\frac{V_{p}}{A_{c}l_{c}}}$$

Here, U is constant for the tangent speed of the compressor. $a_{s}$ is the speed of the sound, $A_{c}$ is compressor’s cross-sectional area, and $V_{p}$ represents plenum volume [26].

The state-space equations of the compressor is described in (5).

$$\dot{x_{1}}=\frac{1}{4B^{2}l_{c}}\left(x_{2}-\phi_{T}\left(x_{1}\right)+d_{\phi }\left(\xi \right)\right)$$

(5) $$\dot{x_{2}}=\frac{1}{l_{c}}\left(\psi_{c}\left(x_{2}\right)-x_{1}-u+d_{\psi }\left(\xi \right)\right)$$

**Figure 7:**
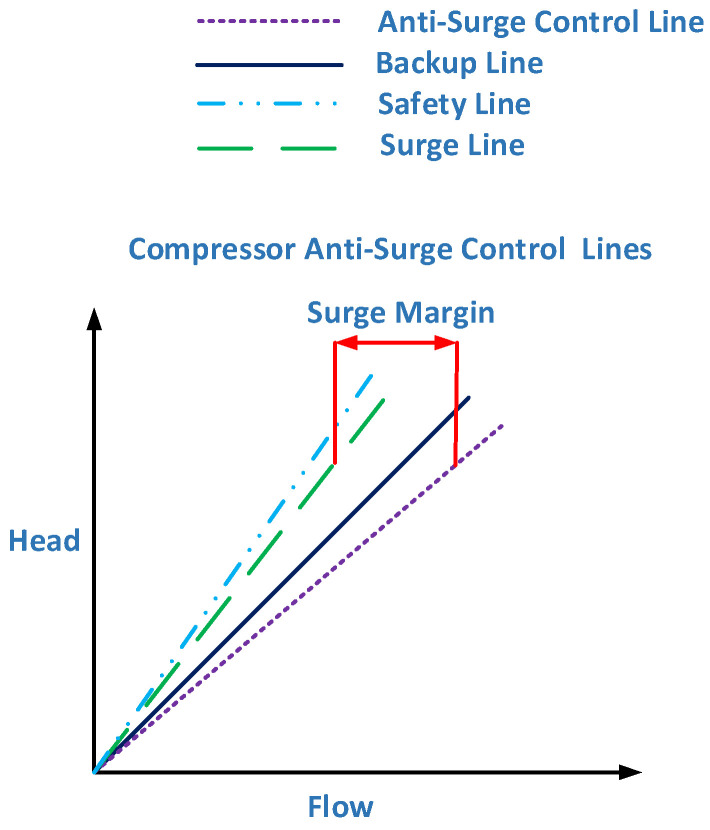
The surge control lines [21].

Different sensors play a vital role in the calculation of the surge. Combining the equations of these sensors, we formulate the virtual values for the AFTCS. The design of an advanced compressor control system is shown in Figure 7, in which the objective is to keep CC to the right of the anti-surge control line. If the operating point crosses this line due to surging conditions, backup lines operate further control actions to open the ASV so that the operating point is driven to the right [21].

**Figure 8:**
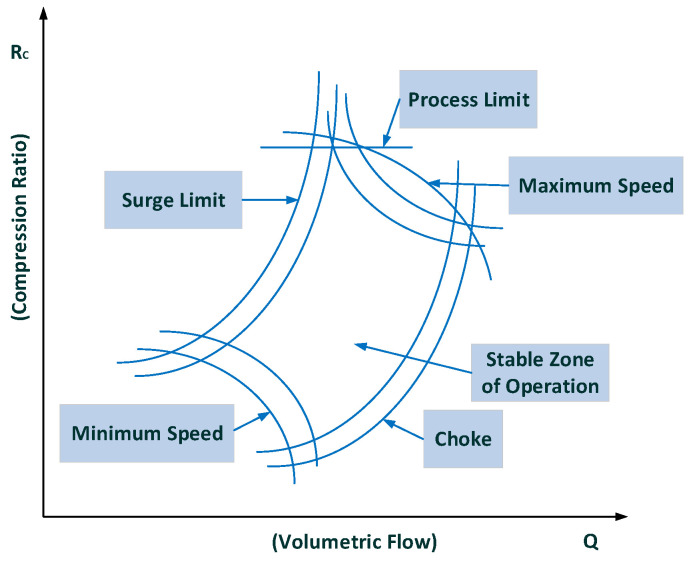
The compressor map [21].

By defining different conditions of compressor low flow limits and maximum power calculations, a compressor map is drawn, as shown in Figure 8, where the stable operating zone of the compressor can be seen at the center.

**Figure 9:**
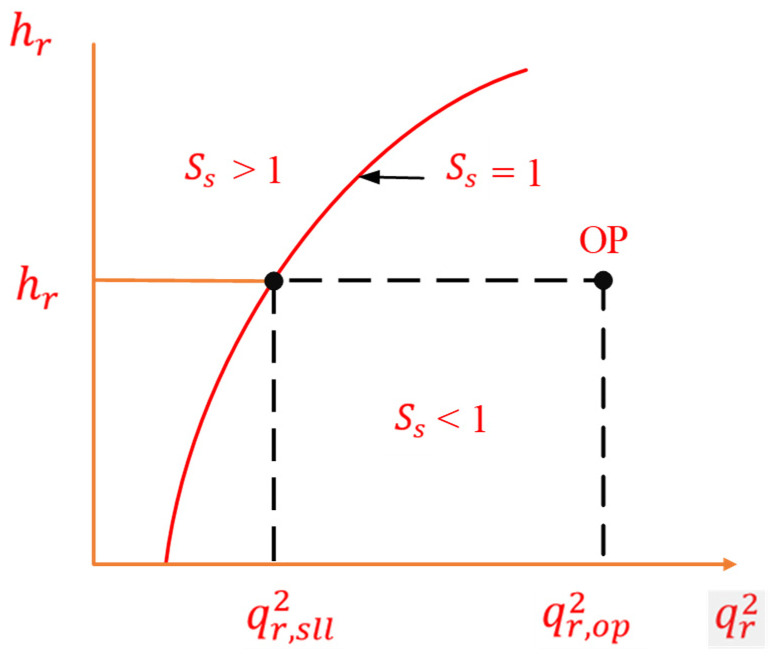
The surge parameter limits [22].

To calculate a controlling parameter, we define a surge parameter (*S_s_*). The calculations for the surge parameter are performed as under [22]:

(6) $$R_{c}=\frac{P_{d}}{P_{s}}$$

(7) $$\sigma =\log\left(\frac{T_{d}}{T_{s}}\right)-\log\left(\frac{P_{d}}{P_{s}}\right)$$

(8) $$h_{r}=\frac{\left(R_{c}^{\sigma }-1\right)}{\sigma }$$

(9) $$q_{r}{}^{2}=\frac{\Delta P_{os}}{P_{s}}$$

(10) $$S_{s}=\frac{q_{r,sll}^{2}}{q_{r,op}^{2}}$$

where *R_c_* is the compression ratio, *P_d_* is discharge pressure, *P_s_* is suction pressure, *T_s_* is suction temperature, *T_d_* is discharge temperature, *σ* is polytrophic head exponent, *h_r_* is reduced pressure head, $q_{r}{}^{2}$ is reduced flow, *ΔP_os_* is the differential pressure across the orifice plate in the flow transmitter, *S_s_* is the surge parameter, $q_{r,sll}^{2}$ is the reduced flow at SLL, and $q_{r,op}^{2}$ is the reduced flow of operating point. Here, the *S_s_* is obtained using Equation (10). Surge parameter elaboration can be observed in Figure 9.

### 1.5. Faults in the Compressor Control System

For a typical process control system, some parameters are crucial for the system’s overall stable behavior. Sensors and actuators are the key elements in a process control system. Generally, sensors are installed at the input of the system and are responsible for providing the first information signal to the controller. Sensors, in the process control system, are generally used for the measurement of temperature, pressure, flow, level, and other varying parameters. These sensors can be of an analog or digital type, depending upon the nature and requirement of the process. The digital sensors send only binary logical signals, upon which the controller takes the decision, while the analog signal usually is in the range of 4–20 mA or 0–10 V (standard signals).

Actuators are generally responsible for the opening and closing of the control valves and are installed at the output of the system. The controller decides according to the inputs and sends an output signal to actuators. Actuators control the valves, which can be digital, i.e., fully opened and fully closed actions, and can be analog for the partial control of the valves. Actuators are the key components in any process control system.

The failure of the sensors and the actuators in the anti-surge system can occur causing a shutdown of the system. An FTC will be required to compensate for the faulty sensors in the ASC systems for service continuity. When any sensor drops out of the system due to fault, the value of the faulty sensor will be replaced by the virtual sensor values, providing the analytical redundancy and the reliability of the system will be increased. For that purpose, an estimated model needs to be run in parallel with the actual plant, from which the healthy value will be obtained to replace the faulty value.

In [27], a sliding mode controller is used to reallocate the actuator position for the application of FTC. A comprehensive review of sliding mode control utilization in FTC is carried out in [28]. In [29], an adaptive design for tracking has been used by utilizing neural networks, to provide the estimated function of the fault using the Lyapunov method. Therefore, by using the adaptive control system, the system efficiency is boosted. Their work is carried out for the nonlinear system only. In [30], FTC is applied with observer-dependent control using a sliding mode approach where the sensor faults, actuator faults, and input noise are handled. Both faults in sensors and actuators are rectified using a sliding mode controller. In [31], altitude tracking is carried out for the aircraft with no need of finding the inertia, which proves to make for a quicker and more efficient system. Additionally, the same technique is used to remove actuator faults. AFTCS implementation can be observed in [32,33,34,35]. In [36], a unified FTC architecture was proposed for the sensors and the actuator based on advanced analytical and hardware redundancies for the sensors of the air-fuel ratio control system of internal combustion engines for achieving greater reliability. In [37], Fuzzy Logic-based AFTCS was proposed for sensor faults of the air-fuel ratio control of internal combustion engines. In [38], a fuzzy-based fault-tolerant controller was made for the robot control mechanism in which the lost signal of the controller was recalculated using two main controllers and the actuator faults were avoided.

In this paper, the main contribution is the implementation of a novel AFTCS to tolerate the failure of the sensors and the actuators in the compressor control system. The AFTCS was built with a dedicated FDI unit to detect and isolate the faulty part as well as replace the faulty value with the virtual redundant value from the observer model running in parallel with the other healthy sensors. Four sensors that play a critical role in the ASC of compressors, namely, the suction temperature, the discharge temperature, the suction pressure, and the discharge pressure, are made faulty one at a time, and then a virtual sensor provides an estimated value for each sensor, respectively. This process is automatically controlled by the controller designed in the HYSYS, which consists of virtual redundant sensor models running in parallel with the system. Hardware redundancy is also added by running a redundant actuator in parallel with the main actuator, and during the fault in the actuator, the controller replaces the faulty actuator with the redundant actuator. The proposed FTC for ASC is novel in the literature and significant for the process industries to design a highly reliable compressor control system that would continue operation despite faults in the sensors and actuators, hence preventing costly production loss.

The further contents of this paper are arranged in this way: the research methodology is elaborated in Section 2, the results and discussions are described in Section 3, and the paper conclusion is presented in Section 4.

## 2. Research Methodology

### 2.1. Implementation of AFTCS

We have implemented AFTCS for the ASC of a CC in this research study. The fault injection unit (FIU) was developed to inject faults manually in the sensors and actuators in the HYSYS using the spreadsheet controller. The function of the FIU is to emulate the fault to make the sensor faulty using a binary switch. When the switch is operated, the fault value of the sensor is fed to the controller instead of the actual; hence, a fault is injected into the system. The fault values are taken close to zero (fail-low), not exactly zero to avoid calculation errors due to the denominator occurrence in the controller calculations.

The advanced split PI+D controller is used to control the surge with a dedicated ASC controller [21], while the AFTCS is implemented in a separate spreadsheet controller. The FDI unit was built to detect and isolate the faulty sensor in the compressor control system. The faulty value is then replaced with the observer/estimator models running in parallel with the other healthy sensors providing analytical redundancy. The parallel system value will then be provided to ASC to replace the faulty sensor value to continue the system and make it fault-tolerant. In this process, the key sensors being used for the ASC are suction pressure, discharge pressure, suction temperature, and discharge temperature.

The working of the observer is described in [1,2]. The state-space representation of the system can be written as follows:

(11) $$\dot{x}=Ax+Bu$$

(12) y=Cx+D

where ‘*x*’, ‘*u*’, and ‘*y*’ denote the states, inputs, and outputs, respectively. The symbols *A*, *B*, *C*, and *D* represent the system matrices of appropriate dimensions.

Let $\bar{x}$ be the estimated state produced by the observer and $\bar{y}$ be the estimated output, then

(13) $$\dot{\bar{x}}=A\bar{x}+B\;u$$

(14) $$\bar{y}=C\bar{x}$$

(15) $$\left(\dot{\bar{x}}-\dot{x}\right)=A\left(\bar{x}-x\right)$$

where $\bar{x}-x=e_{x}$ denotes the error or residual between the estimated and actual state. Additionally,

(16) $$\left(\bar{y}-y\right)=C\left(\bar{x}-x\right)$$

(17) $$\dot{\bar{x}}=A\bar{x}+Bu+L\left(\bar{y}-y\right)$$

where L symbolizes the state feedback gain matrix.

(18) $$\left(\dot{\bar{x}}-\dot{x}\right)=A\left(\bar{x}-x\right)+L\left(\bar{y}-y\right)$$

(19) $$\left(\bar{y}-y\right)=C\left(\bar{x}-x\right)$$

(20) $$\left(\dot{\bar{x}}-\dot{x}\right)=\left(A+LC\right)\left(\bar{x}-x\right)$$

(21) $${\dot{e}}_{x}=\left(A+LC\right)e_{x}$$

(22) $$\left(\bar{y}-y\right)=Ce_{x}$$

The FDI detects the fault by calculating the residual $e_{x}$ and comparing it with the threshold (*ξ*), as follows:

If $e_{x}<\xi$, no fault is present in the sensor

If $e_{x}\geq \xi$, a fault is detected in the sensor and will be replaced by the observer output

The nonlinear observer can be designed for FTC application as:

(23) $$\dot{\bar{x}}\left(t\right)=A\bar{x}\;\left(t\right)+Bu+g\left(\bar{x},\;u,\;t\right)+\bar{L}\left(C\bar{x}-y\right)$$

Let $e_{x}\left(t\right)$ be the error vector

(24) $$e_{x}\left(t\right)\hat{=}\bar{x}\left(t\right)-x\left(t\right)$$

From Equations (21) and (23), we get

(25) $${\dot{e}}_{x}=\left(A-\bar{L}C\right)e_{x}\left(t\right)+\left(g\left(\bar{x},\;u,\;t\right)-g\left(x,\;u,\;t\right)\right)$$

**Lemma** **1:** *Let us consider the equation for a nonlinear system’s observer design*

(26) $$\dot{\bar{x}}\left(t\right)=A\bar{x}+Bu+g\left(\bar{x},\;u,\;t\right)+\bar{L}\left(C\bar{x}-y\right)$$

*where, A, B, and C are matrices; “g” is a function of x, u, and y; and, finally,* $\bar{L}$ *is the feedback gain for the nonlinear observer.*

Let $e_{x}\left(t\right)$ be the error

(27) $$e_{x}\left(k\right)\hat{=}\bar{x}\left(t\right)-x\left(t\right)$$

For a nonlinear system observer, the error equation is

(28) $${\dot{e}}_{x}=\left(A-\bar{L}C\right)e_{x}\left(k\right)+\left(g\left(\bar{x},u,t\right)-g\left(x,u,t\right)\right)$$

The error $e_{x}\left(k\right)$ approaches zero asymptotically if the matrices *R*, *X*, and scalar *µ* exist such that $R=R^{T}>0\;\mathrm{and}\;\mu >0$ to satisfy the following linear matrix inequality (LMI):

(29) $$\left[\begin{array}{cc} RA+A^{T}R+XC+C^{T}X^{T}+\mu \lambda^{2}I & R\\ R & -\mu I \end{array}\right]<0$$

*R* is the reliability of each sensor, and λ denotes its eigenvalue.

Additionally, the observer gain matrix can be selected as follows:

(30) $$\bar{L}=R^{-1}X$$

By considering the following Lyapunov function to prove its derivative to be zero, we can justify the choice of the observer gain matrix:

(31) $$V\left(k\right)=e_{x}^{T}Re_{x}\left(k\right)$$

Now, we will check $\dot{V}\left(x\right)<0\;\forall \;x\epsilon D-\left\{0\right\},$ as described below:

(32) $$\begin{array}{cc} \dot{V}\left(k\right)=e_{X}^{T}(RA\;+ & R\bar{L}C+A^{T}R+C^{T}L^{-T}R)e_{x}+2e_{x}^{T}R\left(g\left(\bar{x},u,k\right)-g\left(x,u,k\right)\right)\\ & \leq e_{X}^{T}\left(RA+R\bar{L}C+A^{T}R+C^{T}L^{-T}R\right)e_{x}+1/\mu e_{x}^{T}R^{2}e_{x}+\mu {\left\|g\left(\bar{x},u,k\right)-g\left(x,u,k\right)\right\|}^{2}\\ & \leq e_{X}^{T}\left(RA+R\bar{L}C+A^{T}R+C^{T}L^{-T}R\right)e_{x}+1/\mu e_{x}^{T}R^{2}e_{x}+\mu \lambda^{2}{\left\|e_{x}\right\|}^{2}\\ & =e_{X}^{T}\left(\left(RA+R\bar{L}C+A^{T}R+C^{T}L^{-T}R\right)+\mu \lambda^{2}I+1/\mu R^{2}\right)e_{x} \end{array}$$

Substituting (30) for (32), we get:

(33) $$\dot{V}\left(k\right)\leq e_{X}^{T}\left(RA+XC+A^{T}R+C^{T}X^{T}+\mu \lambda^{2}I+1/\mu R^{2}\right)e_{x}$$

If the following inequality holds, $e_{x}$ converges asymptotically to zero.

(34) $$\left(RA+XC+A^{T}R+C^{T}X^{T}+\mu \lambda^{2}I+1/\mu R^{2}\right)<0$$

The last equation becomes equivalent to the first equation, which completes the proof.

**Theorem** **1.** *The error* $e_{x}\left(t\right)$ *approaches zero exponentially with the rate* ⲕ/2 *if the matrices R, X, and scalars µ,* ⲕ *exist such that* $R=R^{T}>0\;and\;\mu ,\;ⲕ>0$ *to satisfy the following:*

(35) $$\left[\begin{array}{cc} RA+A^{T}R+XC+C^{T}X^{T}+\mu \lambda^{2}I+ⲕR & R\\ R & -\mu I \end{array}\right]<0$$

To prove this, consider (33) and (35) to obtain

(36) $$\dot{V}\left(t\right)\leq -ⲕe_{x}^{T}Re_{x}=-ⲕV\left(t\right)$$

Hence, we can write

(37) $$V\left(t\right)\leq e_{x}^{T}V\left(0\right)$$

From (42) we obtain

(38) $$\lambda_{min}\left(R\right)\;{\left\|e_{x}\left(t\right)\right\|}^{2}\;\leq \;e^{-kt}\lambda_{max}\left(R\right)\;{\left\|e_{x}\left(0\right)\right\|}^{2}$$

where $\lambda_{min}\;\mathrm{and}\;\lambda_{max}$ are the minimum and maximum eigenvalues of *R*, respectively. Hence, we obtain the following norm:

(39) ‖ex(t)‖≤λmax (R)λmin(R) ‖ex(0)‖ e−kt/2

Coming back to the residual equation:

(40) $$r\left(t\right)\hat{=}\|C\bar{x}\left(t\right)\;-\;y\left(t\right)\|$$

(41) r(t)≤λmax (R)λmin(R) C(0)‖ex(0)‖ e−kt/2

(42) $$\left\|C\left(0\right)\|\|e_{x}\left(0\right)\|\approx \|r\left(0\right)\right\|$$

Finally, we can come up with the following criteria for the fault detection of a sensor fault

(43) r (t){≤ λmax (R)λmin(R) ‖r(0)‖ e−kt2, there is no fault>λmax (R)λmin(R) ‖r(0)‖ e−kt2, there is a fault

Using Equations (44)–(47), an estimated value of one sensor is obtained in terms of the other sensors. This scenario provides a parallel running model of each sensor. The sensor value will be replaced by this estimated model value when a fault has occurred in the system [18].

(44) $$P_{s}=\frac{P_{d}}{R_{c}}$$

(45) $$P_{d}=R_{c}\;\cdot \;P_{s}$$

(46) $$T_{s}=\frac{T_{d}}{R_{c}{}^{n}}$$

(47) $$T_{d}=T_{s}\;\cdot \;R_{c}{}^{n}$$

**Figure 10:**
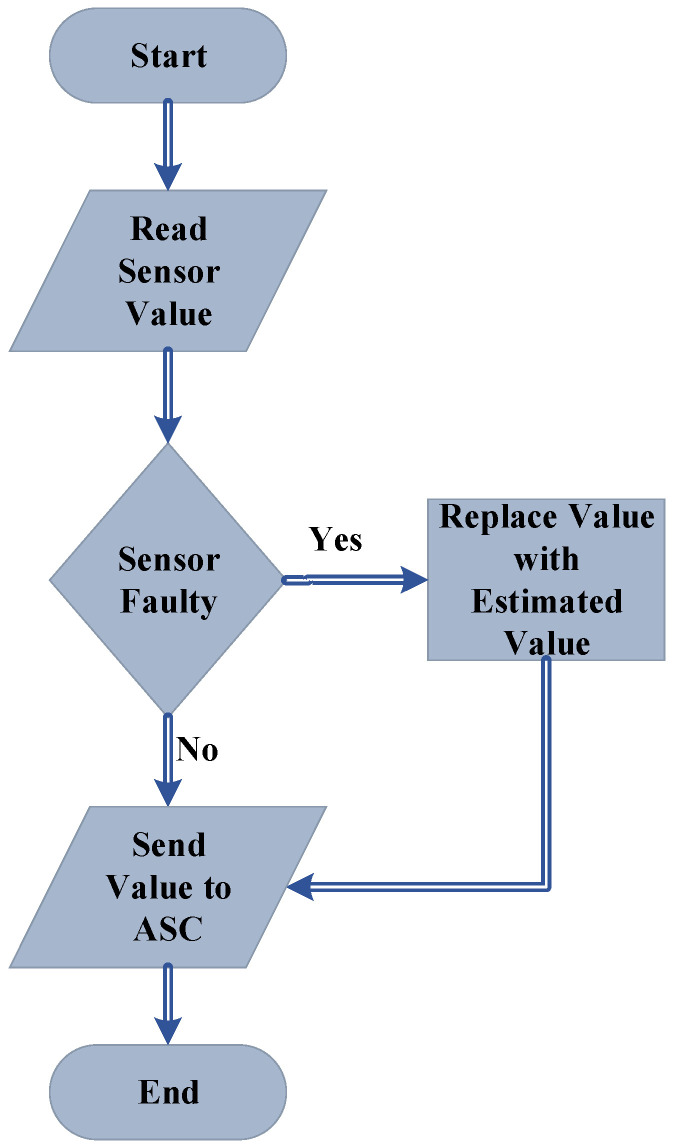
The fault-tolerant anti-surge control flowchart.

The proposed scheme can be described in a flowchart as shown in Figure 10. The sensor value is replaced according to the given scheme as described in Figure 10; the actual process will not be disturbed, and the sensor fault will be tolerated. It should also be noted that this scheme only bypasses the fault, while the actual fault is still there and the sensor needs to be replaced. The FTC ensures the system continues its process regardless of the fault occurrence.

### 2.2. HYSYS Model for Centrifugal Compressor

The centrifugal compressor dynamic model is implemented in the HYSYS software. A compressor is attached along with its assembly with the suction and discharge valves and the main recycle valve. The stream is introduced using the suction valve, and then the liquid is removed from the fluid using a scrubber; after that, the gas is entered into the compressor. After the discharge end of the compressor, a cooler is placed to reduce the temperature of the gas. When the surge occurs, the ASV is opened fully and the gas is recycled to avoid the surge [17]. The HYSYS model used in this study was first verified with practical gas compressor station parameters. Actual values from the gas station were compared with the HYSYS values, and the percentage (% age) error was calculated to confirm the accuracy and validity of the results. The comparison of important parameters of the HYSYS model and gas compressor station values is shown in Table 1.

It can be seen that all important parameters provide the percentage (% age) error between simulation and practical values within acceptable bounds. This confirms the accuracy and validation of simulation results.

The assumptions of this research contain the consideration of a low fault (fail-low) value for all sensors during the fault rather than a high fault value, and there is no surge in the system when the fault has occurred. The limitations of the research include the interdependency of redundant sensors, i.e., only one sensor failure is considered at one time as the redundant sensor values are calculated using other healthy sensors. Moreover, only complete failure type faults in sensors and actuators are considered by ignoring the system’s faults. This research does not take into account partial faults or unstructured uncertainties.

## 3. Results and Discussion

### 3.1. Fault Tolerance in Suction Pressure

**Figure 11:**
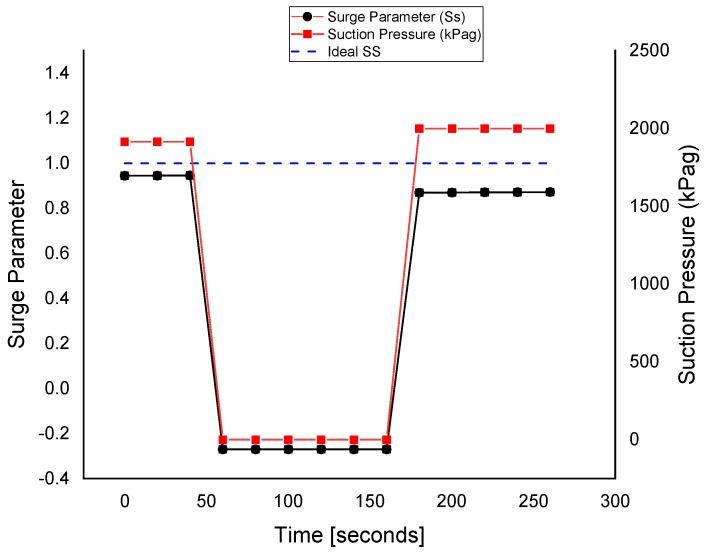
The fault tolerance in the suction pressure sensor.

The suction pressure sensor is made faulty with FIU, as shown in Figure 11. At about the 60 s of the simulation, the fault is injected into the *P_s_* sensor; at that time, the fault tolerance is kept out of the system to differentiate between the faulty and fault-tolerant conditions. At about the 160 s of the simulation, the FTC is turned on and the value of the sensor is replaced with that of the estimated model running in parallel with the FDI unit. It can be observed that the value of the surge parameter also deviates during the fault occurrence, and it regains its value using the estimated value of the sensor, provided by the equation models.

The fault is tolerated for the suction pressure sensor, and this improves the system reliability and prevents unnecessary flow across the ASV. When the wrong signal of the suction pressure sensor is fed to the controller, the ASV is opened by the ASC. After that, the wrong or faulty signal is interrupted by the FTC and replaced by a healthy value, i.e., the estimated value obtained from the observer. This provides an analytical redundancy for the *P_s_* sensor.

**Figure 12:**
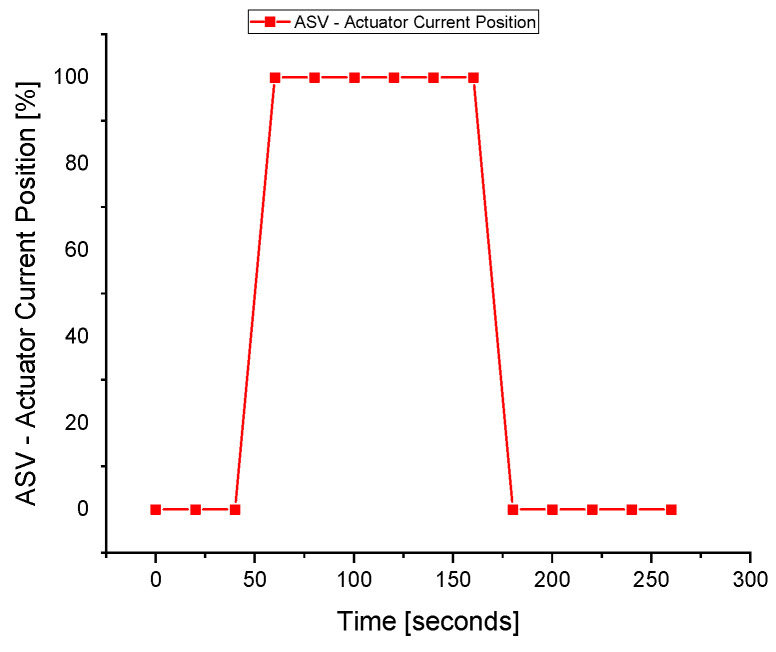
The actuator response during a fault in the *P_s_* sensor.

The ASV response during the fault execution for the suction pressure sensor is shown in Figure 12. The flow of the fluid across the ASV during the fault is a waste of energy, especially when the wrong signal of the surge is fed to the controller due to a fault in the sensors. The valve operates when the faulty *P_s_* signal is sent to the ASC at about the 60 s. The fault is tolerated at about 160 s when the valve is fully closed, and the system returns to normal despite the fault occurrence in the *P_s_* sensor.

### 3.2. Fault Tolerance in Discharge Pressure Sensor

**Figure 13:**
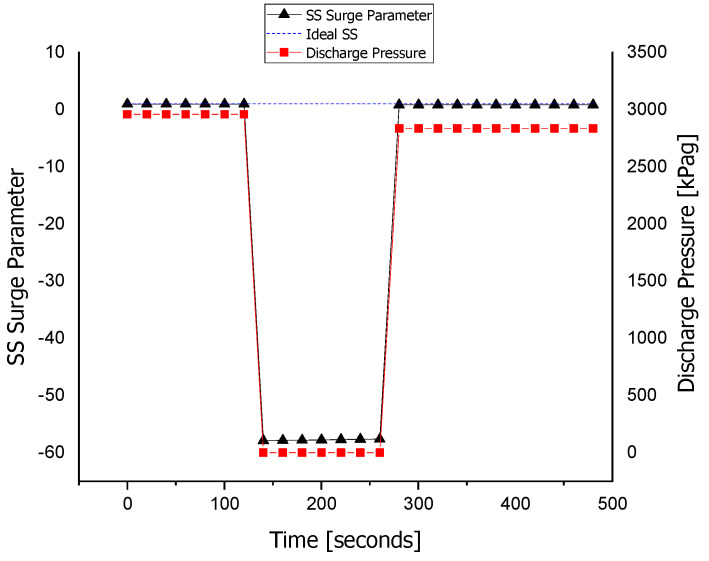
The fault tolerance in the discharge pressure sensor.

The discharge pressure sensor is made faulty with FIU, as shown in Figure 13. At about the 140 s of the simulation, the fault is injected into the *P_d_* sensor; at that time, the fault tolerance is kept out of the system to differentiate between the faulty and the fault-tolerant conditions. At about the 260 s of the simulation, the FTC is turned on and the value of the sensor is replaced with that of the estimated model running in parallel with the FDI unit. It can be observed that the value of the surge parameter also deviates during the fault occurrence, and it regains its value using the estimated value of the sensor that is provided by the equation models.

The fault is tolerated for the discharge pressure sensor, and this improves the system reliability and prevents unnecessary flow across the ASV. When the wrong signal of the discharge pressure sensor is fed to the controller, the ASV is opened by the ASC. After that, the wrong or faulty signal is interrupted by the FTC and replaced by a healthy value, i.e., the estimated value obtained from the observer. This provides an analytical redundancy for the *P_d_* sensor.

**Figure 14:**
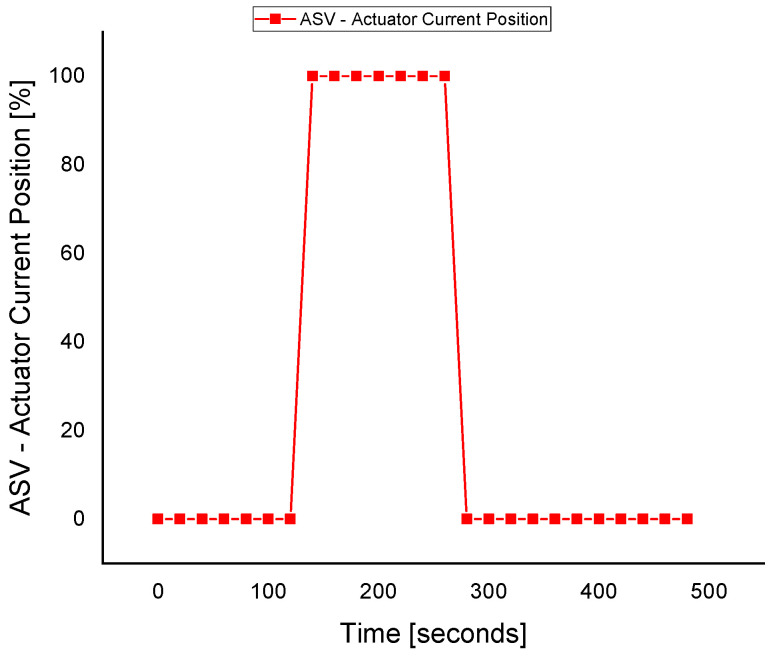
The actuator response during a fault in the *P_d_* sensor.

The ASV response during the fault execution for the discharge pressure sensor is shown in Figure 14. The flow of the fluid across the ASV during the fault is a waste of energy, especially when the wrong signal of the surge is fed to the controller due to the fault in the sensors. The valve operates when the faulty *P_d_* signal is sent to the ASC at about 140 s. The fault is tolerated at about the 260 s, where the valve is fully closed, and the system returns to normal despite the fault occurrence in the *P_d_* sensor.

### 3.3. Fault Tolerance in Suction Temperature Sensor

**Figure 15:**
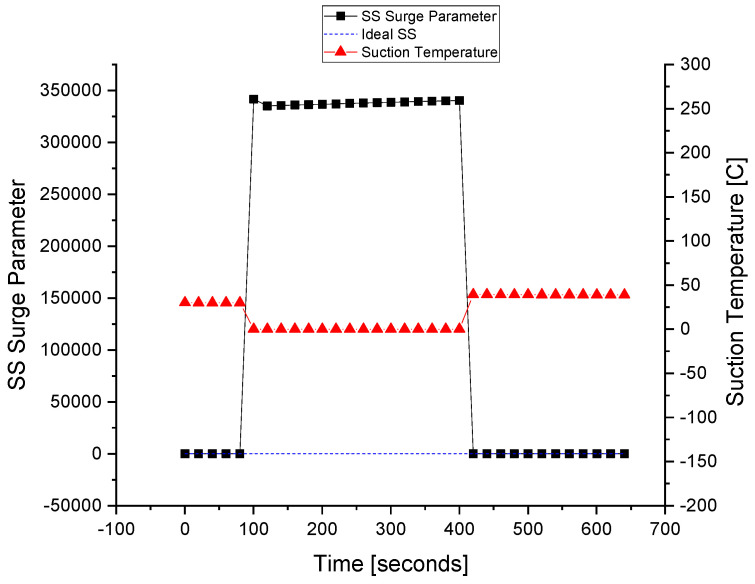
The fault tolerance in the suction temperature sensor.

The suction temperature sensor is made faulty with the FIU, as shown in Figure 15. At about 80 s of the simulation, the fault is injected into the *T_s_* sensor at that time the fault tolerance is kept out of the system to differentiate between the faulty and fault-tolerant conditions. At about the 420 s of the simulation, the FTC is turned on and the value of the sensor is replaced with that of the estimated model running in parallel with the FDI unit. It can be observed that the value of the surge parameter also deviates during the fault occurrence, and it regains its value using the estimated value of the sensor that is provided using the equation models.

The fault is tolerated for the suction temperature sensor, and this improves the system reliability and prevents unnecessary flow across the ASV. When the wrong signal of the suction temperature sensor is fed to the controller, the ASV is opened by the ASC. After that, the wrong or faulty signal is interrupted by the FTC and replaced by a healthy value, i.e., the estimated value obtained from the observer.

**Figure 16:**
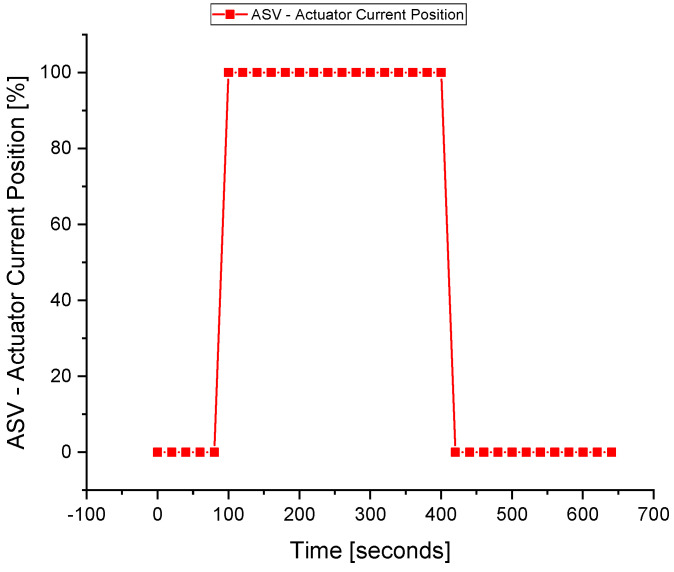
The actuator response during a fault in the *T_s_* sensor.

The ASV response during the fault execution for the suction temperature sensor is shown in Figure 16. The flow of the fluid across the ASV during the fault is a waste of energy, especially when the wrong signal of the surge is fed to the controller due to the fault in the sensors. The valve operates when the faulty *T_s_* signal is sent to the ASC at about 80 s. The fault is tolerated at about 420 s when the valve is fully closed, and the system returns to normal despite the fault occurrence in the *T_s_* sensor.

### 3.4. Fault Tolerance in Discharge Temperature Sensor

**Figure 17:**
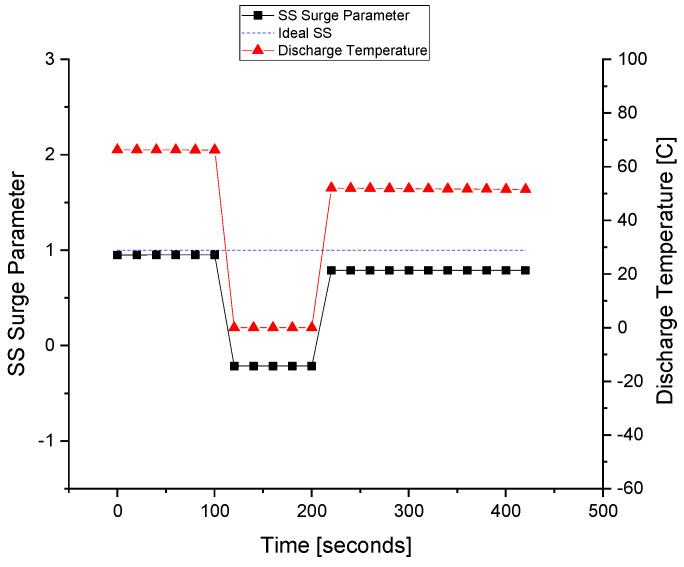
The fault tolerance in the discharge temperature sensor.

The discharge temperature sensor is made faulty with FIU, as shown in Figure 17. At about the 110 s of the simulation, the fault is injected into the *T_d_* sensor; at that time, the fault tolerance is kept out of the system to differentiate between the faulty and fault-tolerant conditions. At about the 210 s of the simulation, the FTC is turned on and the value of the sensor is replaced with that of the estimated model running in parallel with the FDI unit. It can be observed that the value of the surge parameter also deviates during the fault occurrence, and it regains its value using the estimated value of the sensor that is provided by the equation models.

The fault is tolerated for the discharge temperature sensor, and this improves the system reliability and prevents unnecessary flow across the ASV. When the wrong signal of the discharge temperature sensor is fed to the controller, the ASV is opened by the ASC. After that, the wrong or faulty signal is interrupted by the FTC and replaced by a healthy value, i.e., the estimated value obtained from the observer.

**Figure 18:**
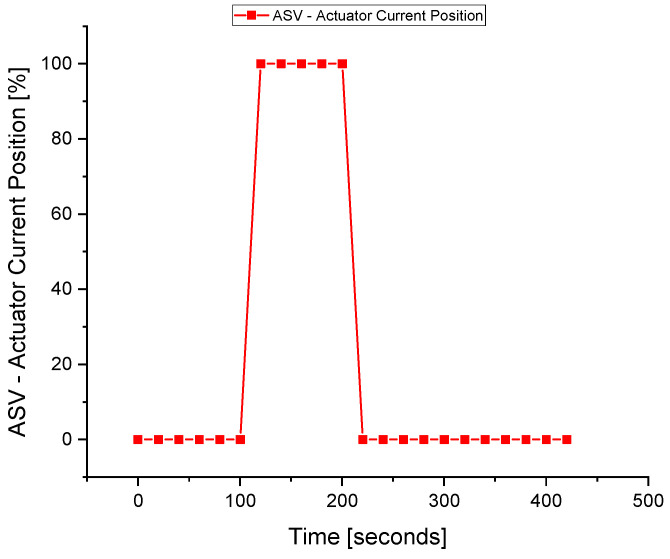
The actuator response during a fault in the *T_d_* sensor.

The ASV response during the fault execution for the discharge temperature sensor is shown in Figure 18. The flow of the fluid across the ASV during the fault is a waste of energy, especially when the wrong signal of the surge is fed to the controller due to the fault in the sensors. The valve operates when the faulty *T_d_* signal is sent to the ASC at about 110 s. The fault is tolerated at about the 210 s when the valve is fully closed, and the system returns to normal despite the fault occurrence in the *T_d_* sensor.

### 3.5. Fault Tolerance in Actuators

The failure of the actuator in the field can cause greater problems. Unlike sensors, the actuator needs to be replaced when a fault has occurred. Therefore, a redundant ASV-2 is installed in parallel with the existing ASV-1. When the actuator of the first valve becomes faulty, the parallel valve comes into operation.

**Figure 19:**
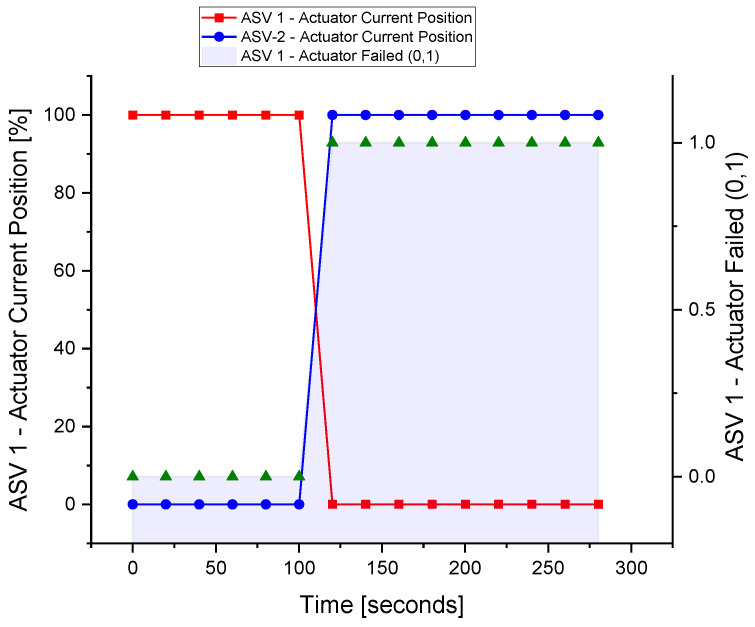
The fault tolerance in the actuator.

The actuator of ASV-1 is made faulty in HYSYS by using the built-in feature of the actuator failed condition. Once the actuator fails, the controller reads the failed value and the second valve actuator works immediately to provide hardware redundancy as in Figure 19.

The proposed research work is novel and has not been found in the literature so far; therefore, we were not able to compare our results with any other existing works.

## 4. Conclusions

In this paper, a novel AFTCS was proposed for the sensor and actuator faults of the anti-surge control system of a centrifugal compressor. The AFTCS was built with a dedicated FDI unit to detect and isolate the faulty part as well as replace the faulty value with the virtual redundant value from the observer model running in parallel with the other healthy sensors. The analytical redundancy was developed from the mathematical modeling of the sensors to provide the estimated values to the controller in the case of the failure of the actual sensor. Dual hardware redundancy was proposed for the anti-surge valve. The simulation results of the proposed FTC for the ASC in HYSYS reveal that the system continued to operate in the event of faults in the sensors and actuators. The proposed FTC for the ASC is novel in the literature and significant for the process industries to design a highly reliable compressor control system that would continue operation despite faults in the sensors and actuators, hence preventing costly production loss.

Future work may involve FTC implementation with other advanced intelligent controllers such as fuzzy logic control and artificial neural networks. System identification can also be implemented for the better development of the redundant models of the sensors.

## Figures and Tables

**Table 1:** A comparison of the CC HYSYS model with practical values.

Description	Field Values	HYSYS Values	Percentage	Units
			Error (%)	
Suction Temperature	33.5	33.81	0.93	degC
Suction Pressure	1534	1529	0.33	kPag
Discharge Temperature	98.8	88.3	10.63	degC
Suction Flow	13.7	13.42	2.04	kPag
Discharge Pressure	2982	2925	1.91	kPag
Compressor Speed	7744	7744	0.00	RPM
Suction Throttle	99.77	100	0.23	%
ASV Position	0	0	0.00	%

## Data Availability

This statement is not required in our paper as we don’t report any data.

## Abbreviations

CC	Centrifugal Compressor
FTC	Fault-Tolerant Control
AFTCS	Active Fault-Tolerant Control System
ASC	Anti-Surge Control
ASV	Anti-Surge Valve
FIU	Fault Injection Unit
FTC	Fault-Tolerant Control
OP	Operating Point
PFTCS	Passive Fault-Tolerant Control System
SCL	Surge Control Line
SLL	Surge Limit Line
TMR	Triple Modular Redundancy
*∆P_os_*	Differential Pressure
*h_r_*	Reduced Head Pressure
*K_c_*	Proportional Gain
*P_d_*	Discharge Pressure
*P_s_*	Suction Pressure
$q_{r}^{2}$	Reduced Flow
*R_c_*	Compression Ratio
*S_s_*	Surge Parameter
*T_d_*	Discharge Temperature
*T_i_*	Integral Reset Time
*T_s_*	Suction Temperature
*σ*	Polytrophic Head Exponent
$q_{r,sll}^{2}$	Reduced flow at SLL
